# Supplementary material for: Diagnostic Performance of Multi-Detector Computed Tomography Arthrography and 3-Tesla Magnetic Resonance Imaging to Diagnose Experimentally Created Articular Cartilage Lesions in Equine Cadaver Stifles
Source: Animals (Basel). 2023 Jul 14;13(14):2304. doi: 10.3390/ani13142304 (PMC10376593; doi:10.3390/ani13142304)
Supplement: Supplementary file 1 [file animals-13-02304-s001.zip › animals-2427689-supplementary.pdf]

Table S1. Magnetic resonance imaging sequences and parameters used in the present study.

Sequence	T2W_TSE_ Sag	T2W_TSE_ Cor	T2W_TSE_ Tra	3D_T2W_ HR	3D_PDW_ SPAIR	T1W_VISTA_ SPAIR
TR	4557	4674	3737	1300	1200	350
TE	80	80	80	257	194	19
Flip angle	90	90	90	90	90	90
Echo train length	14	12	20	85	42	24
NEX	1	1	1	1	1	1
FOV	250x250x181 mm	230x202x188 mm	220x220x221 mm	220x202x300 mm	200x250x300 mm	250x199x300 mm
Frequency direction	FH	RL	RL	AP	FH	AP
Fat suppressed	No	No	No	No	No	No
Slice thickness	3 mm	3 mm	3 mm	0.55 mm	0.7 mm	0.7mm
Gap width	3 mm	3 mm	3 mm	-0.3 mm	0 mm	-0.35 mm
Number of slices	55	57	67	382	286	571
Time to run (min)	7.45	6.13	6.14	8.28	7.52	7.46

Abbreviations: Sag, sagittal; Cor, Coronal (dorsal); Tra, Transversal, HR, High-resolution; TR, time to repeat; TE, time to echo; NEX, Number of excitations; FOV, field of view; FH, foot to head (distoproximal); RL, right to left; AP, anterior to posterior (craniocaudal).
